# Rice Bran-Based Bioplastics: Effects of Biopolymer Fractions on Their Mechanical, Functional and Microstructural Properties

**DOI:** 10.3390/polym14010100

**Published:** 2021-12-28

**Authors:** María Alonso-González, Manuel Felix, Alberto Romero

**Affiliations:** 1 Departamento de Ingeniería Química, Facultad de Química, Universidad de Sevilla, 41012 Sevilla, Spain; 2 Departamento de Ingeniería Química, Escuela Politécnica Superior, Universidad de Sevilla, 41011 Sevilla, Spain; mfelix@us.es (M.F.); alromero@us.es (A.R.)

**Keywords:** bioplastics, rice bran, rice bran oil, valorization, starch, injection molding

## Abstract

Rice bran is an underutilized by-product of rice production, containing proteins, lipids and carbohydrates (mainly starches). Proteins and starches have been previously used to produce rice bran-based bioplastics, providing a high-added-value by-product, while contributing to the development of biobased, biodegradable bioplastics. However, rice bran contains oil (18–22%), which can have a detrimental effect on bioplastic properties. Its extraction could be convenient, since rice bran oil is becoming increasingly attractive due to its variety of applications in the food, pharmacy and cosmetic industries. In this way, the aim of this work was to analyze the effect of the different components of rice bran on the final properties of the bioplastics. Rice bran refining was carried out by extracting the oil and fiber fractions, and the effects of these two procedures on the final properties were addressed with mechanical, functional and microstructural measures. Results revealed that defatted rice bran produced bioplastics with higher viscoelastic moduli and better tensile behavior while decreasing the water uptake capacity and the soluble matter loss of the samples. However, no significant improvements were observed for systems produced from fiber-free rice bran. The microstructures observed in the SEM micrographs matched the obtained results, supporting the conclusions drawn.

## 1. Introduction

Environmental pollution derived from conventional plastics produced from fossil resources has become a global concern. Consequently, the production of environmentally sustainable materials as an alternative is drawing the attention of the scientific community [1,2,3]. Great effort has been made to develop biodegradable plastics from renewable natural resources with the aim of producing biodegradable materials that resemble the behavior of fossil-based polymers. It is estimated that, between 1950 and 2015, 8.3 billion tons of plastic were produced worldwide and only 21% were recycled or incinerated, the remaining 79% accumulated in landfills and surrounding areas [4]. In addition, conventional plastics are not biodegradable, thus they remain in the environment for many years, physically breaking into smaller particles occurs, releasing microplastics in landfills and marine environments, entering food chains and, consequently, animal bodies, eventually causing different diseases [5,6].

In this context, bioplastics, which can be either biobased or biodegradable, appear as a promising alternative to replace or at least reduce the extensive use of conventional plastics and their harmful waste [7,8]. Biodegradable plastics are usually made from biopolymers. Furthermore, biopolymers derived from renewable sources, such as animals or plants, can play an essential role in overcoming the challenges derived from the depletion of oil resources and the environmental problems related to the increasing use of petroleum-based plastics. These biopolymers can be natural fibers, cellulose, polysaccharides, proteins, lipopolysaccharides, etc. [9,10].

Natural polymers such as polysaccharides (starch, cellulose, pectin, hemicellulose) and proteins (casein, zein, gluten, gelatin) are generally able to form intramolecular and intermolecular interactions and cross-linking between polymeric constituents, forming a semi-rigid three-dimensional network [11]. Furthermore, these biopolymers are present in wastes and by-products from the agro-food industry. For example, rice (*Oryza sativa* L.) is the major cereal crop grown in the world [12] and is generally processed by shelling and polishing to remove the bran from the grain prior to commercialization. Although rice bran constitutes about one-tenth of the rice weight, it is still underutilized. It is a low-added-value by-product mainly used for animal feeding or as an organic fertilizer [12]. However, the proteins and starches present in rice bran have previously been used to develop rice bran-based bioplastics; their mechanical and functional properties depend on the processing parameters and composition [13,14]. This valorization turns rice bran into a high-added-value by-product, a new raw material, such as gluten or soybean flours [15,16], that can be used to produce biobased, biodegradable plastic, benefiting both the environment and the producing companies. However, there are no studies that evaluate the effect of the different fractions of rice bran on their mechanical and functional properties.

Furthermore, rice bran is emerging as a potential by-product of rice processing also as a result of the increasing demand for rice bran oil (RBO). Rice bran contains 18–22% oil, similar to other edible vegetable oil sources such as soybean (15–20%) or tung (16–18%) [17]. Rice bran oil is unique among edible vegetable oils due to its composition (i.e., fatty acids, phenolic compounds and vitamin-E) [18]. Among the health benefits of RBO, it reduces oxidative stress and hypertension, has anti-cancer and anti-diabetic activities, can act as anti-inflammatory or anti-allergic agent, etc. In this way, it has a variety of food and non-food applications in pharmacy and cosmetics [18,19,20].

In this context, this research work aimed to analyze the effect of oil extraction, which can have its own valorization route, on the properties of defatted rice bran-based bioplastics. In addition, the fibers were also extracted to evaluate the influence of the different biopolymer fractions on the properties of the bioplastics obtained. In this way, different systems were obtained from virgin rice bran, defatted rice bran, and fiber-free rice bran, each of them plasticized with a mixture of water with either glycerol or sorbitol. By these means, the plasticizer effect was also analyzed for the different active matters employed. The other processing parameters, raw materials proportion, mixing temperature and injection pressure and temperature, were kept constant, selected based on previous studies. Through these means, the effects of both the biopolymer fractions and the selected plasticizer were successfully analyzed, achieving a better understanding of the mechanisms involved in the development of protein- and starch-based bioplastics.

## 2. Materials and Methods

### 2.1. Materials

Vaporized indica rice bran (RB) was obtained from Herba Ingredients (San José de la Rinconada, Spain). Deionized-grade water, sorbitol and glycerol were employed as plasticizers. Both sorbitol and glycerol were provided by PANREAC S.A. (Barcelona, Spain). All other reagents were supplied by Sigma-Aldrich (St. Louis, MI, USA).

### 2.2. Preparation of Defatted Rice Bran (DRB) and Fiber-Free Rice Bran (FRB)

DRB was prepared by suspending sieved RB (<500 μm) in hexane (1:10 *w/v*). The mixture was vigorously stirred at room temperature for 24 h and, after this time, it was centrifuged at 5000 rpm for 10 min. The supernatant containing both hexane and lipids was carefully separated from the solid fraction, which was dried in a fume hood to remove any residual hexane. This process was carried out twice, ensuring that >90% of the lipids were removed.

Fiber removal was carried out following the methodology used by Singh et al. [12] by first soaking DRB in deionized grade water (1:40 *w/v*) for 2 h. The pH was then adjusted to 9.5 using a NaOH solution and the mixture was stirred at room temperature for 1 h. After this time, the slurry was sieved through a 125 μm mesh to separate the fibers and the remaining mixture was centrifuged. The supernatant (containing some soluble protein) was discarded and the solid fraction was washed with distilled water and lyophilized.

### 2.3. Chemical Composition

The chemical composition of RB was already characterized by Alonso-González et al. [13]. The approximate composition of DRB and FRB flours was determined following the approved methods of A.O.A.C. [21]. The water content was determined by mass difference after placing 3 g of sample in a conventional oven (Memmert B216.1126, Schwabach, Germany) at 105 °C for 24 h. The lipid content was quantified using the Soxhlet extraction method [22], where hexane was used as a solvent in contact with the sample. The lipids were dragged in subsequent cycles until the whole lipid content was removed and quantified by mass difference. The ash content was determined by heating a small amount of sample at 550 °C in a muffle furnace (Hobersal HD-230, Barcelona, Spain) for 5 h in air atmosphere. The sample was then cooled to room temperature in a desiccator before being weighed again to calculate the mass difference. Protein content was determined as% N × 6.25 [23] using a LECO TRUSPEC CHNS-932 nitrogen microanalyzer (Leco Corporation, St. Joseph, MI, USA). Finally, the starch and fiber content in the RB sample was determined by analytical methods in an external laboratory and it was assumed that their ratios were constant after defatting (i.e., DRB system) while their contents in the FRB were determined by mass difference.

### 2.4. Sample Preparation

Samples were prepared according to the methodology followed by Alonso-González et al. [14]. In this way, the active matter (RB, DRB and FRB) was introduced along with plasticizers into a HAAKE POLYLAB QC mixer-rheometer (ThermoScientific, Waltham, MA, USA), equipped with counter-rotating rotors, obtaining homogeneous blends. Two different plasticizers, glycerol (G) and sorbitol (S), were evaluated in combination with water (W) for each system. All blends contained 55% active matter and 45% total plasticizer, maintaining the proportion of 2:1 water-G/S. The proportions were selected according to previous studies [13,14]. The blends were mixed at 200 rpm and 80 °C for 1 h. The systems are identified with the corresponding active matter (RB, DRB and FRB) and the plasticizer used (G or S). In this way, a system developed from defatted rice bran using a mixture of water and sorbitol would be DRBS.

Once mixed, the doughs were kept inside a desiccator until the moisture content was adequate for further processing (between 10 and 40 wt.%). The final moisture content, calculated during the drying process following the A.O.A.C. methods, varied for each system. The doughs were finally processed by injection molding using a Haake pneumatic piston injection molding equipment (MiniJet ThermoScientific, Waltham, MA, USA) to obtain the bioplastic samples. The temperatures for the injection cylinder and the mold were 50 °C and 150 °C, respectively. The injection pressure was 500 bar, which was applied for 15 s, while the post-injection time and pressure were 200 s and 500 bar, respectively. These conditions were selected according to previous studies [13,14]. By these means, rectangular probes (60 mm × 10 mm × 1 mm) were obtained and employed for mechanical, functional and microstructural characterization.

### 2.5. Bioplastics Characterization

#### 2.5.1. Dynamic Mechanical Thermal Analysis (DMTA)

DMTA tests were carried out with a DMA850 rheometer (TA Instruments, New Castle, DE, USA) on the rectangular probes using the film clamp in tension mode. First, the linear viscoelastic range was determined by strain sweep tests. Subsequently, a strain within the linear viscoelastic range was selected for the frequency sweep tests (between 0.01 and 20 Hz) performed at room temperature and the temperature ramp tests (between −10 and 160 °C). Temperature tests were carried out at constant frequency (1 Hz) at the heating rate of 5 °C/min. By these means, the elastic modulus (E′), viscous modulus (E″) and loss tangent (tan δ = E″/E′) were obtained for the whole studied range.

#### 2.5.2. Tensile Tests

Tensile tests were performed with RSA3 equipment (TA Instruments, New Castle, DE, USA) according to a modification of the ISO 527-2 method for the tensile properties of plastics [24] using rectangular probes and measuring three replicates. Stress-strain curves were obtained for all evaluated systems and Young’s modulus, maximum stress and deformation at break were successfully determined with a deformation rate of 1 mm/min at room temperature.

#### 2.5.3. Water Uptake Capacity and Soluble Matter Loss

Water uptake capacity (WUC) was measured according to the ASTM D570 method [25] using one-third of the rectangular samples mentioned above, that is, probes measuring 20 mm × 10 mm × 1 mm. The samples were subjected to a dehydrothermal treatment in a conventional oven at 50 °C (Memmert UN 55, Schwabach, Germany) for 24 h to determine the dry weight (*Initial dry weight*). Subsequently, they were immersed in distilled water and weighted after 24 h of immersion (*Wet weight*). Finally, they were frozen at −40 °C before lyophilization at −80 °C and vacuum atmosphere (0.125 bar) using a LyoQuest freeze-dryer with a Flask M8 head (Telstar, Barcelona, Spain) and subsequently weighted (*Final dry weight*). According to the methodology used, *WUC* and soluble matter loss (*SML*) were determined by the following equations:

(1) $$WUC\;\left(\%\right)\;=\;\frac{Wet\;weight-Initial\;dry\;weight}{Initial\;dry\;weight}\cdot 100$$

(2) $$SML\;\left(\%\right)\;=\;\frac{Initial\;dry\;weight-Final\;dry\;weight}{Initial\;dry\;weight}\cdot 100$$

#### 2.5.4. Scanning Electron Microscopy (SEM)

Selected bioplastics samples after water uptake and subsequent freeze-drying (1 mm thick) were observed by SEM examination, showing the micrographs their microstructural appearance. The equipment used was a ZEISS EVO microscope (Carl Zeiss Microscopy, White Plains, NY, USA) at a voltage of 10 kV and a magnification of 500×. These samples were previously coated by Pd/Au sputtering (13 nm) using a Leica AC600 metalizer. Finally, the porosity of the samples was calculated using ImageJ software.

### 2.6. Statistical Analyses

At least three replicates of each measurement were carried out. Statistical analyses were performed using t-test and one-way analysis of variance (ANOVA) (significance value ρ < 0.05) using the STATGRAPHICS 18 software (Statgraphics Technologies, Old Tavern Rd, The Plains, VA, USA). Standard deviations from some selected parameters were calculated. Significant differences are indicated by different letters, that is, all mean values labeled with the same letter did not show significant differences.

## 3. Results

### 3.1. Chemical Composition

The chemical composition of this variety of rice bran is similar to those obtained by different authors in their research, the fiber and starch content being those with the highest differences depending on the variety [26,27]. The effects of lipid and fiber extractions on the chemical composition of RB are gathered in Table 1. First, the lipid extraction produced a DRB system with only 1.8% lipids, which caused a proportional increase in the rest of the components as happened in a similar study directed by [12]. Thus, the chemical characterization revealed a composition of 15.9% moisture, 13.4% ashes, 16.8% proteins and 27.9 and 24.2% fiber and starch, respectively. Previous studies revealed that the protein content consists mainly of variable fractions of glutelin, globulin, albumin, and prolamin, while the starch content is the mixture of two biopolymers: amylose and amylopectin [12,28]. However, the fiber removal process caused the lipid fraction to increase proportionally again to 5.8%. Consequently, the rest of the fractions also increased containing 16.8% moisture, 24.6% ashes, and 34.1% starch, leaving the remaining of 3.0% fibers. The only exception would be the protein content, which appeared to decrease to 15.7% due to the removal of some soluble protein during the fiber extraction procedure.

### 3.2. Dynamic Mechanical Thermal Analyses (DMTA)

**Figure 1:**
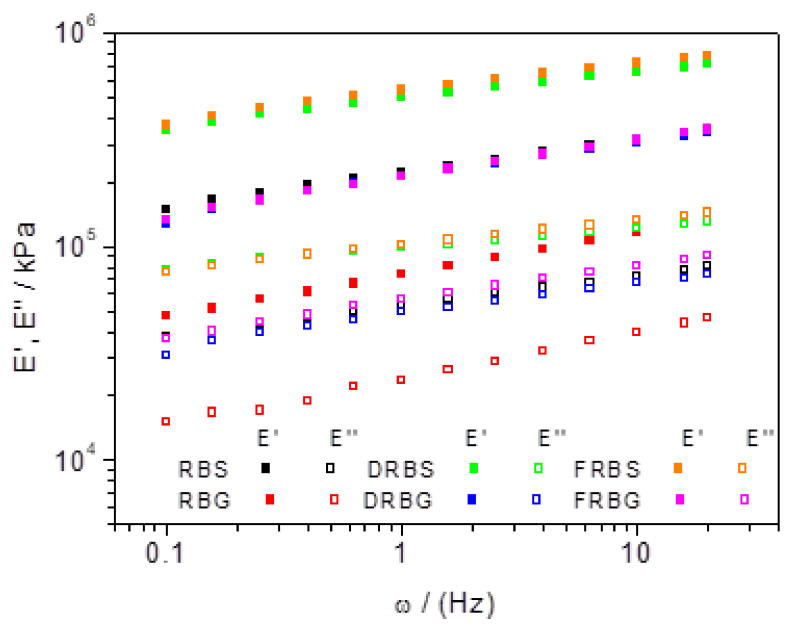
Frequency sweep tests performed between 0.1 and 20 Hz of the different studied systems (RBS, RBG, DRBS, DRBG, FRBS, and FRBG) carried out at room temperature.

The frequency sweep tests of rice bran-based bioplastics are shown in Figure 1. As can be seen, E′ is higher than E″ for all processed systems, which confirms that the samples processed by the injection molding process exhibit mainly elastic behavior. At the same time, the values obtained for the viscoelastic moduli present a certain frequency dependence, increasing as the frequency increases for all studied systems. Regarding the active matter, the lowest recorded values were observed for RB systems, processed with either glycerol or sorbitol as plasticizers (that is, RBG and RBS, respectively). However, this analysis shows that the lipid and fiber extractions resulted in bioplastics with better rheological behavior than the virgin RB, with the viscoelastic moduli increasing for all DRB- and FRB-based systems, with no significant differences between them. In this way, the values of the viscoelastic moduli overlay for the DRBS and FRBS systems, as well as for the DRBG and FRBG. Finally, it can be observed that sorbitol produced bioplastics with higher viscoelastic moduli than those processed with glycerol as a plasticizer. In fact, the two glycerol systems with better rheological behavior, DRBG and FRBG, present practically similar E′ and E′ with respect to RBS.

**Figure 2:**
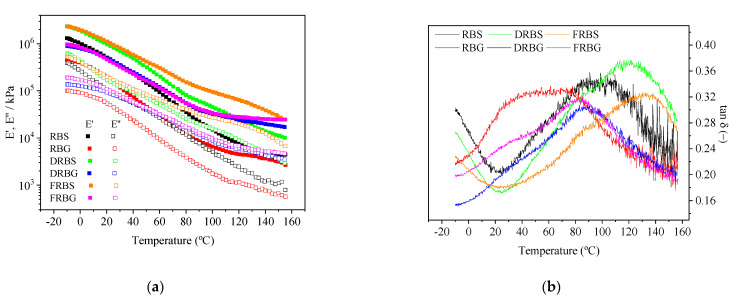
Temperature sweep tests between −10 and 160 °C performed on the different evaluated systems at 1 Hz; (**a)** Elastic (E′) and viscous (E″) modulus (**b**) Loss tangent (tan δ).

Figure 2a,b show the temperature dependence of viscoelastic moduli (E′ and E″) and tan δ, respectively. As can be seen in Figure 2a, all systems exhibit the same behavior, where, again, the elastic component is always above the viscous one within the whole frequency range studied. Furthermore, in all cases, both E′ and E″ decrease with increasing temperature; the difference between them is the abruptness of this drop towards the end of the experiment. First, the recorded values followed the same trend as in the previous case: the two virgin systems (RBS and RBG) presented the lowest E′ and E″ values, while the two extraction methods appear to enhance their rheological behavior. In this way, the DRBS and FRBS systems are above the RBS one, and the DRBG and FRBG systems are above the RBG samples. However, although in Figure 1 both extraction methods seem to give similar bioplastics, the DRB and FRB samples differ from each other in this case, since the FRBS and FRBG underwent a less accused drop of the viscoelastic moduli with temperature being above DRBS and DRBG during the last part of the studied range (T > 90 °C). In addition, the same tendency as in Figure 1 is maintained with the sorbitol systems which exhibited higher E′ and E″ than the glycerol ones; however, in this case, there is a great difference in their behavior depending on the plasticizer used. Thus, the viscoelastic moduli of the glycerol systems seem to stabilize at the end of the experiment, maintaining their values, whereas for the effect of the plasticizer used (sorbitol or glycerol), since the sorbitol-based bioplastics continue to decrease for the whole temperature range studied.

Figure 2b shows the evolution of the loss tangent with temperature. The observed maximum values correspond to the glass transition temperature (T_g_) of the plasticized RB, DRB and FRB [29]. In this way, the influence of the biopolymer fraction as well as the plasticizer used on the T_g_ of the systems can be analyzed. First, it can be observed that glycerol led to a Tg earlier than sorbitol. In this way, the T_g_ of the RBG appeared around 50 °C, while for the RBS is around 100 °C. A similar behavior was shown by DRBG and FRBG, whose transition temperatures were nearly 80–90 °C, although their equivalent sorbitol systems underwent the glass transition at 120–130 °C, approximately. In addition, the effect of the biopolymer fractions can be observed in both the widths of the peaks and the higher T_g_ with respect to the virgin RB. In this way, the two extraction procedures, oil and fiber removal, led to narrower peaks with respect to virgin RB. The RBG and RBS systems showed wider transitions. Additionally, it is possible to distinguish the beginning of another peak (at the lowest temperatures studied) for the samples plasticized with the water-sorbitol mixture that is related to the glass transition of the plasticizer mix [30]. For the samples plasticized with water-glycerol, this peak would be expected at lower temperatures.

### 3.3. Tensile Tests

**Figure 3:**
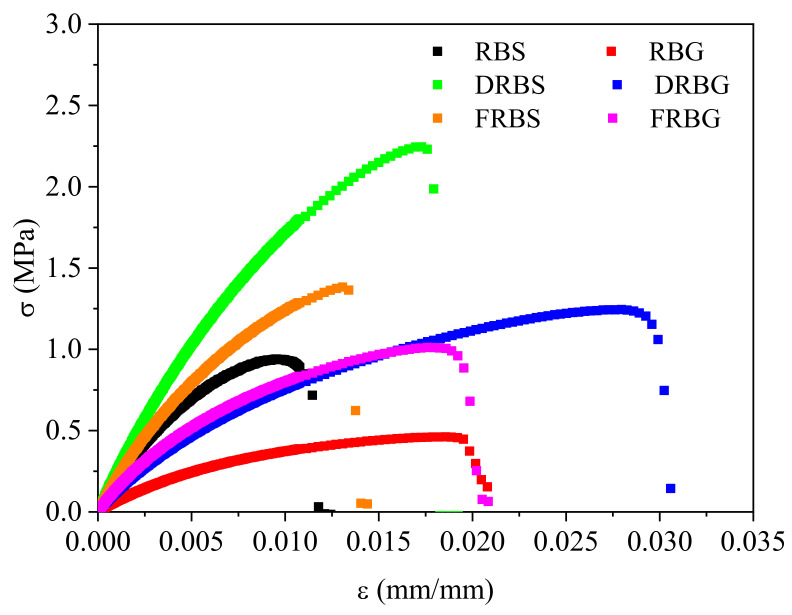
Stress-strain curves obtained for the different evaluated systems at 1 mm/min and room temperature.

Figure 3 shows the stress-strain curves obtained for the different samples. As can be seen, both extraction procedures enhanced the tensile response of the obtained bioplastics. In this way, the response corresponding to the RBS samples is below those of DRBS and FRBS, and the DRBG and FRBG systems are above the RBG curve. Moreover, when comparing the three different active matters employed in this study, the samples with better mechanical properties are those obtained from the defatted rice bran, since the fiber-free samples did not induce further improvements in the mechanical properties. On the other hand, it can be observed that the sorbitol systems exhibited higher stiffness and withstood higher stresses, while the glycerol-based probes presented improved elasticity, achieving, by these means, greater elongations.

The three parameters obtained from the strain-stress curves (that is, Young’s modulus (YM), maximum stress (MS), and deformation at break (DB)) are gathered in Table 2, which allows for a more accurate evaluation of the tensile properties. First, the values obtained for the YM improved for the treated RB samples and for the systems containing sorbitol. Therefore, the lowest value is associated with the RBG sample 53 ± 1 MPa, followed by its two treated systems, namely DRBG and FRBG, which did not show significant differences exhibiting 104 ± 2 and 106 ± 9 MPa, respectively. The systems plasticized with sorbitol exhibited improved stiffness compared to the previous ones, beginning with the similar values recorded for the RBS and FRBS systems (156 ± 6 and 171 ± 17 MPa), which were exceeded by the DRBS, obtaining the highest value (227 ± 11 MPa). MS values followed a similar trend with the lowest and highest values presented by RBG and DRBS, respectively. The second-lowest was the FRBG system, followed closely by the RBS and DRBG systems and, finally, the FRBS system. The last parameter, that is, DB, differed from the other two. Although defatted samples induced better elongations (DRBS and DRBG), no significant differences were observed for fiber-free systems. The RBS and FRBS values did not show significant differences, either RBG nor FRBG. In addition, the elastic deformation of the bioplastics was enhanced by glycerol instead of the improvements observed in the other parameters for the sorbitol-containing samples.

### 3.4. Water Uptake Capacity and Soluble Matter Loss

**Figure 4:**
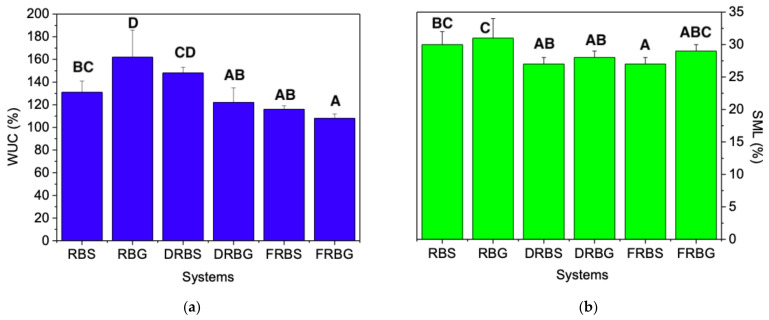
(**a**) Water uptake capacity (WUC) and (**b**) soluble matter loss (SML) of the different evaluated systems. Different letters above columns indicate significant differences (*p* < 0.05).

The water uptake capacity (WUC) and soluble matter loss (SML) of the obtained systems are shown in Figure 4a,b, respectively. This figure indicates that the removal of lipids and fibers has a detrimental effect on WUC, since treated samples exhibited lower absorption capacities (especially those without fiber). The only exception is the DRBS sample, with a value of 148 ± 5%, while its native sample showed a lower value (131 ± 10%), although the decreasing trend was again followed by the FRBS sample, with only 116 ± 3%. This drop in the absorption capacities was more progressive for glycerol-containing samples (RBG, DRBG, and FRBG systems). Thus, the highest value is associated with the RBG sample, with 162 ± 24%, followed by the DRBG sample, with 122 ± 13%, and finally the FRBG sample, with 108 ± 4%, which are the lowest results of all evaluated systems. The WUC values obtained are similar to those observed by López-Castejón et al. [30] for albumen/tragacanth-based bioplastics. 

However, the effects were less remarkable for the SML values. It seems that higher WUC is associated with higher SML, although there were no significant differences. In this way, the lowest values were those of the FRBS and DRBS systems with 27 ± 1%, followed by the DRBG and FRBG systems, with 28 ± 1% and 29 ± 1%, respectively. Finally, the two systems with the highest losses are the RBS and RBG systems, with 30 ± 2% and 31 ± 3%, respectively.

### 3.5. Scanning Electron Microscopy (SEM)

**Figure 5:**
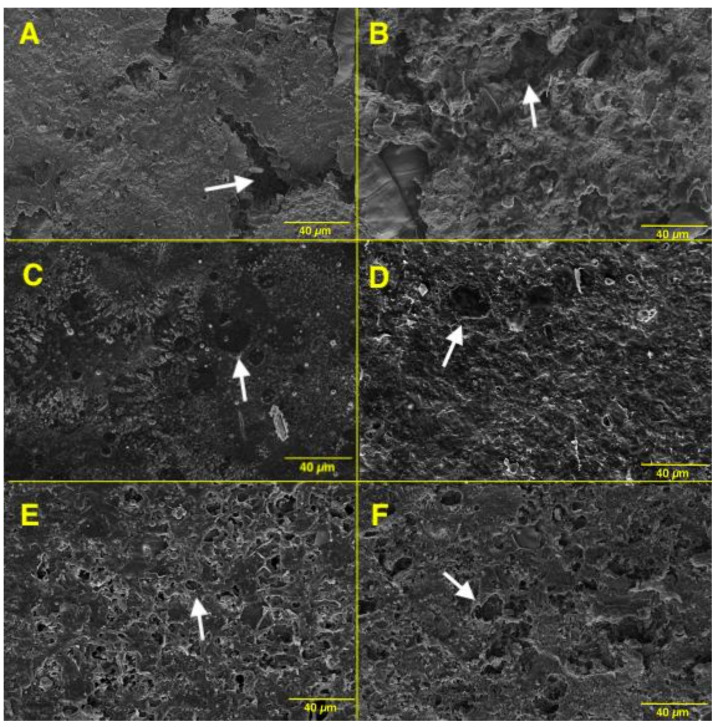
SEM micrographs of the different lyophilized bioplastics observed (**A**) RBS; (**B**) RBG; (**C**) DRBS; (**D**) DRBG; (**E**) FRBS; (**F**) FRBG.

Figure 5 shows the SEM micrographs obtained for the different bioplastics. Figure 5A,B correspond to the samples obtained with the raw RB containing sorbitol and glycerol, respectively. These two samples present microstructures with great flaws, large pores and cracks with no continuous surface, showing 15.8 and 25.8% porosity, respectively. On the contrary, the effect of employing treated rice bran is perfectly noticeable. In this way, the defatted rice bran produces more continuous surfaces with smaller pores, which present a more homogeneous distribution, as can be observed in Figure 5C,D. Although the pores are smaller, the total porosity increases for these two samples to 40.4% for DRBS and to 39.4% for DRBG. On the other hand, the effect is more pronounced in the last two micrographs, i.e., E and F, corresponding to the fiber-free samples, which again exhibit homogeneous surfaces with smaller pores, maintaining the total porosity in 38.4 and 38.1%, respectively. Finally, when the two plasticizers used are compared, both sorbitol and glycerol seem to produce similar porosities.

## 4. Discussion

### 4.1. Chemical Composition

The chemical composition of RB, DRB, and FRB gathered in Table 1 allows analyzing the efficiency of the extraction methods. The lipid extraction was successfully carried out, decreasing the fat content from 22.8% for the RB sample to 1.8% for the DRB sample. Therefore, within 48 h after suspension in hexane, 92.11 ± 0.6% lipids were extracted at room temperature. Although the lipid extraction could be optimized using the Soxhlet device, removing most of the fat content in hours, the sample would reach higher temperatures, which could imply certain thermal modifications (i.e., earlier gelatinization) that are avoided with the methodology used. Note that lipids have been reported to act as plasticizer, increasing the mechanical properties of the final material generated [31]. Moreover, the hydrophobic character of lipids may also affect the water uptake capacity of the bioplastics obtained, this will be discussed in Section 4.4. With respect to the removal of the fibers, the methodology employed allowed the removal of the fiber fraction along with some soluble protein. This removal of fiber must affect the final properties of the bioplastic generated. It should be noticed that fiber used as filler can reinforce the structure of polymers. However, an excessive amount also may affect the mechanical properties of the final bioplastics [32]. The different systems studied will allow evaluating the effect of the fiber fraction on the bioplastic formed.

### 4.2. Dynamic Mechanical Thermal Analyses (DMTA)

Figure 1 shows the frequency sweep tests of predominantly elastic probes, indicating a frequency dependence. This dependence was already observed in previous studies conducted on RB-based bioplastics [13]. This frequency dependence has been previously related for uncross-linked polymers of high molecular weight [33], which is the case of these polymers, where the biopolymer chains (i.e., proteins or carbohydrates), are not specifically linked to each other. Thus, the systems plasticized with water/sorbitol (RBS, DRBS and FRBS) led to higher E′ and E″ than water/glycerol ones (RBG, DRBG and FRBG), which according to the “lubricating” theory, the glycerol has a higher lubricating effect [34]. With respect to the active matter, the removal of the lipid fraction in the DRBS and DRBG-based bioplastics produced probes with improved rheological properties, that is, higher viscoelastic moduli. Thus, these results confirm that rice bran oil could act as a plasticizer, which increases the biopolymer chain mobility, leading to reduced viscoelastic moduli. Lipid removal appears to be beneficial for rheological behavior, since the viscoelastic moduli increased. The improvement in rheological behavior could also be attributed to the relative increase in the number of proteins and starches, that is, the active matter that produces most biopolymers. In this way, although fiber removal could have a similar effect, increasing total interactions, fibers are known to act as reinforcement in the biopolymeric matrix [26], therefore, the two effects counteract each other, leaving bioplastics without significant improvements in the rheological properties, as can be observed in the E′ and E″ values of the FRBS and FRBG samples. In this experiment, the DRB and FRB systems maintained a similar behavior.

Some of the effects described above can also be observed in Figure 2a. First, the removal of the lipids again led to enhanced rheological behavior, with the DRBS and DRBG systems exhibiting viscoelastic moduli above the RBS and RBG systems, respectively, which are associated with a higher proportion of active matter, producing more interactions among the biopolymer chains. Moreover, the lack of RB oil reduced the mobility of the biopolymer chain as a result of its plasticizer role. However, although no significant differences were observed between the DRB and FRB systems when evaluating the frequency dependence, fiber removal enhanced the rheological behavior with temperature, especially for the FRBS samples, which were above the DRBS samples above room temperature, while the FRBG system is only superior to the DRBG towards the end of the temperature interval studied. Since fibers do not contribute to creating significant interactions in protein- and starch-based biopolymers [35,36], the lack of fibers allows creating a more compact structure with better behavior with temperature for higher protein and starch proportions. Finally, in terms of the plasticizer effect, although sorbitol contributes to the production of bioplastics with higher viscoelastic moduli, E′ and E″, these systems experienced a more longer temperature dependence than the systems containing glycerol (which even stabilized above 100 °C until the end of the experiment). This behavior was previously observed in preliminary studies (results not published yet).

In addition, analysis of the loss tangent (tan *δ*) behavior was performed in order to assess the effect of the two extraction methods and the plasticizer used on the glass transition temperature of the bioplastic obtained. Figure 2b reveals that glycerol induced an earlier transition compared to the sorbitol-containing system, indicating a higher plasticizer efficiency for glycerol, which was also deduced from the biopolymer chain mobility above-mentioned. This effect is also reflected in the delayed peaks deduced at the lowest temperatures for the sorbitol-based systems. This higher plasticizer effect of glycerol was also observed when these compounds plasticized whey-based films [37]. The narrow peaks associated with the T_g_ of the DRB and FRB bioplastic using either glycerol or sorbitol as the plasticizer might be related to the purer composition obtained with the two extraction methods followed. As is well-known, purer substances undergo the glass transition in small temperature ranges, while complex compounds exhibit wider transition ranges [38]. In addition, the two extraction procedures exhibited higher T_g_ compared to the virgin RB, which might be due to the lack of oil in their composition, which also reflects its role as plasticizer. Moreover, this plasticizer effect is also observed in the

### 4.3. Tensile Tests

The results collected in Figure 3 and Table 2 confirm the beneficial effects of lipid removal on the mechanical properties of the final bioplastics. In this way, the plasticizer behavior of rice bran oil was lost, while, the proportion of proteins and starches increased, leading to stiffer, more resistant and more elastic samples (i.e., higher E, σ and ε for the DRBS and DRBG samples). This effect has been previously reported when for crayfish-PCL composites when increasing the active matter, which also led to an increase in the tensile parameters [39]. As for the fiber content, its removal also resulted in improved tensile behavior compared to virgin rice bran (RBS and RBG samples), the results for the FRBS and FRBG systems are below those of the defatted ones (DRBS and DRBG). In this case, as previously described, although there was a higher protein and starch content, the reinforcing effect of the fibers was lost and the samples obtained showed poorer tensile-strength properties. With respect to the plasticizer effect of sorbitol and glycerol, it seems that glycerol forms a closer mixture with the active matter, having a more efficient plasticizer effect and producing more elastic but less stiff samples. On the other hand, as a solid material, sorbitol can also act as a filler, not achieving such high elongations but leading to very stiff and resistant samples [40]. These results are in agreement with those obtained from DMA tests, confirming the different roles played by each plasticizer (glycerol or sorbitol). Although the tensile tests assume that the deformation of the material is related to its elasticity, whereas the DMA tests consider that the response is a combination of the elastic and viscous components, both assumptions reach the same conclusion for the plasticizing effect. Thus, this agreement between oscillatory and continuous deformation test has been also found for synthetic polymers such as epoxy foams [41].

### 4.4. Water Uptake Capacity and Soluble Matter Loss

The detrimental effect on WUC and the lower SML associated with defatted and fiber-free samples may be related to the higher proportions of proteins and starches present in these systems. In these samples, the interactions within the active matter were favored in greater proportions, leading to samples with high physical integrity (low soluble matter loss) that hold their structure during water immersion, although with a lower tendency to absorb and retain water. As can be seen in Section 3.4, the effect is observed for the DRBS and DRBG samples, but it was more pronounced in the FRBS and FRBG samples. Regarding the effect of the plasticizer, glycerol seems to produce bioplastics with higher WUC and, consequently, higher SML, as previously indicated, both parameters are related [42]. The effect of the plasticizer can also be related to the higher hydrophilic character of the glycerol, this higher affinity to water favors its penetration, and it also can justify the higher values obtained for the SML parameter, since once solubilized, it can be released in the medium, where the concentration difference is the driving force [43,44]. Moreover, it should be also noted that rice-bran oil can be extracted with both polar (i.e., acetone) and non-polar solvents (e.g., hexane, chloroform) [45]. Although we are not assuming that water extracts rice bran oil, it seems that polar solvent may interact with rice bran oil, facilitating water diffusion through the biopolymer structure, which is required for water uptake [46].

### 4.5. Scanning Electron Microscopy (SEM)

The microstructures observed in Figure 5 can be related to the results obtained in the previous sections regarding mechanical properties and the WUC. In this sense, the improved mechanical properties associated with the purified samples (i.e., the defatted rice bran, Figure 5C,D, and fiber-free rice bran, Figure 5E,F), correspond to more homogeneous microstructures with smaller pores and cracks that also present a more continuous distribution compared to those of Figure 5A,B. Thus, the higher homogeneity found in the micrographs evidenced a lower water uptake, since probe swelling created fewer pores and cracks when more active matter was present. The higher interactions led to enhanced mechanical properties observed. Thus, systems that usually have higher mechanical properties generally have a detrimental effect on water uptake [13], which was also obtained in this study, where the higher porosities observed in the DRB and FRB samples were not related to higher water absorption. Moreover, the glycerol-based samples (Figure 5B,D,F) also seem to exhibit more pores than those obtained with sorbitol (Figure 5A,C,E), which can be related to the above-mentioned of glycerol compared to sorbitol. Higher probe porosity after water uptake as glycerol content increases was previously observed for bioplastics [47].

## 5. Conclusions

This work evidences the beneficial effect of the removal of the lipid fraction on the mechanical properties of bioplastic samples. The lipids imply a certain plasticizing effect, which is lost in the DRB, also leading to a higher proportion of active matter and consequently to better rheological and tensile properties. The higher amount of active matter was later reflected in the WUC of the probes generated. Where also was observed the hydrophilic character of glycerol since it was released in a larger extend than sorbitol (SML values). However, although the removal of the fibers produces a higher proportion of active matter, the reinforcing effect of fibers is eliminated, which causes either a detrimental or no significant effect on the final properties. The only exception is the rheological behavior at high temperatures that was enhanced for these last samples. Finally, among the two plasticizers used, glycerol exhibited a higher plasticizing efficiency, leading to a lower glass transition temperature and producing bioplastics with higher deformability. On the contrary, the sorbitol-containing systems exhibited higher stiffness and maximum stress values.

Furthermore, the chemical characterization of the DRB and FRB revealed great quantities of ash content, which could also be removed via dialysis to obtain an optimized and pure biopolymer. In this way, there are still many modifications and parameters to be evaluated to develop suitable rice bran-based bioplastics. In addition, according to their mechanical and functional properties, RB-based bioplastics could be selected as promising candidates for the substitution of conventional fossil plastics in certain applications in the future.

## Figures and Tables

**Table 1:** Chemical composition of the different active matters employed: rice bran (RB), defatted rice bran (DRB) and fiber-free rice bran (FRB).

Composition	RB	DRB	FRB
Moisture (%)	12.5 ± 5.0	15.9 ± 5.0	16.8 ± 3.4
Ashes (%)	10.5 ± 0.3	13.4 ± 0.5	24.6 ± 0.5
Lipids (%)	22.8 ± 1.3	1.8 ± 0.6	5.8 ± 1.6
Proteins (%)	13.2 ± 0.5	16.8 ± 0.5	15.7 ± 1.0
Fiber (%)	22.0 ± 1.0	27.9 ± 1.0	3.0 ± 1.0
Starches (%)	19.0 ± 1.0	24.2 ± 1.0	34.1 ± 1.0

**Table 2:** Young’s modulus (YM), maximum stress (MS) and deformation at break (DB) of the different evaluated systems obtained from the stress-strain curves.

System	YM (MPa)	MS (MPa)	DB (mm/mm)
RBS	156 ± 6 ^C^*	1.00 ± 0.10 ^FG^	1.26 ± 0.18 ^J^
RBG	53 ± 1 ^A^	0.42 ± 0.04 ^E^	2.03 ± 0.13 ^K^
DRBS	227 ± 11 ^D^	2.30 ± 0.30 ^I^	1.82 ± 0.20 ^K^
DRBG	104 ± 2 ^B^	1.17 ± 0.03 ^GH^	3.04 ± 0.26 ^L^
FRBS	171 ± 17 ^C^	1.33 ± 0.06 ^H^	1.36 ± 0.23 ^J^
FRBG	106 ± 9 ^B^	0.90 ± 0.09 ^F^	2.11 ± 0.13 ^K^

* Different superscript letters within the column indicate significant differences (*p* < 0.05). Significant differences are indicated by different letters, all mean values labeled with the same letter did not show significant differences.
